# Comparing microbiotas in the upper aerodigestive and lower respiratory tracts of lambs

**DOI:** 10.1186/s40168-017-0364-5

**Published:** 2017-10-27

**Authors:** Laura Glendinning, David Collie, Steven Wright, Kenny M. D. Rutherford, Gerry McLachlan

**Affiliations:** 1 The Roslin Institute and Royal (Dick) School of Veterinary Studies, University of Edinburgh, Edinburgh, Midlothian EH25 9RG UK; 2 Animal Behaviour and Welfare, Animal and Veterinary Sciences Research Group, SRUC, West Mains Rd., Edinburgh, Midlothian EH9 3JG UK

**Keywords:** Lung, Microbiota, Sheep, Lambs, Oropharynx, Rumen, 16S

## Abstract

**Background:**

Recently, the importance of the lung microbiota during health and disease has been examined in humans and in small animal models. Whilst sheep have been proposed as an appropriate large animal model for studying the pathophysiology of a number of important human respiratory diseases, it is clearly important to continually define the limits of agreement between these systems as new concepts emerge. In humans, it has recently been established that the lung microbiota is seeded by microbes from the oral cavity. We sought to determine whether the same was true in sheep.

**Results:**

We took lung fluid and upper aerodigestive tract (oropharyngeal) swab samples from 40 lambs (7 weeks old). DNA extraction was performed, and the V2-V3 region of the 16S rRNA gene was amplified by PCR then sequenced via Illumina Miseq. Oropharyngeal swabs were either dominated by bacteria commonly associated with the rumen or by bacteria commonly associated with the upper aerodigestive tract. Lung microbiota samples did not resemble either the upper aerodigestive tract samples or reagent-only controls. Some rumen-associated bacteria were found in lung fluids, indicating that inhalation of ruminal bacteria does occur. We also identified several bacteria which were significantly more abundant in lung fluids than in the upper aerodigestive tract swabs, the most predominant of which was classified as *Staphylococcus equorum*.

**Conclusions:**

In contrast to humans, we found that the lung microbiota of lambs is dissimilar to that of the upper aerodigestive tract, and we suggest that this may be related to physiological and anatomical differences between sheep and humans. Understanding the comparative physiology and anatomy underlying differences in lung microbiota between species will provide a foundation upon which to interpret changes associated with disease and/or environment.

**Electronic supplementary material:**

The online version of this article (10.1186/s40168-017-0364-5) contains supplementary material, which is available to authorized users.

## Background

The use of 16S ribosomal RNA (rRNA) gene sequencing has facilitated the study of difficult to culture, low biomass microbial communities present in the lower respiratory tract. The impact of the lung microbiota on human health is a rapidly growing area of research. In order to understand this impact, it is important to also understand the lung microbiota dynamics during health and to include healthy controls in disease studies. To achieve this, the majority of previous studies have relied on human volunteers.

However, many individuals are hesitant to participate in research bronchoscopy due to the perceived inconvenience and a fear of complications [1], despite the low risk involved. Mice and rats have been used to explore the relationship between the lung microbiota and airway inflammation [2–4], microbiota at different body sites [5], the environment [6], acute lung injury [7] and antibiotic [8] and corticosteroid exposure [9]. However, rodents are of limited use when exploring spatial or longitudinal lung microbiota dynamics due to their small lung size. Recognising the utility of large animal models in this regard, and the anatomical and immunological relevance of sheep as models [10–13], our group has previously used this species to explore the changes in the lung microbiota upon *Pseudomonas aeruginosa* infection [14] and to explore the spatial variability present within the healthy lung [15].

Subclinical microaspiration of pharyngeal secretions is a feature of health and this can contribute to the lung microbiota composition [16], and the microbiome of the human lungs more closely resembles that of the mouth than that of the nose or the lower gastrointestinal tract [17]. It is not yet known whether the same relationship holds for species other than humans. In ruminating sheep, where the oropharynx is exposed to ruminal contents on a frequent basis, one would anticipate that lung microbiota would similarly reflect this influence. In this paper, we find that the presence of rumen-like bacteria in the upper aerodigestive tract is correlated with changes in the lung microbiota and rumen-type bacteria are present in lamb lungs. We also identify bacteria which are more indicative of the lungs than the oropharynx, indicating that the presence of the sheep lung microbiota is not merely due to passive diffusion of microbes from the upper aerodigestive tract.

## Methods

### Animals and sampling

Scottish Mule X Suffolk lambs (20 males and 20 females, unweaned), raised on pasture from 48 h after birth, were used in this study. These lambs were part of a study on the animal welfare implications of prenatal stress which was approved by Scotland’s Rural College’s (SRUC) Animal Experiments Committee and was conducted under Home Office licence. All lambs were raised by their dams, and prior to euthanasia, their only food sources were ewe’s milk and pasture. At 7 weeks old (mean age = 48.8 days ± 0.8 standard deviation (SD); mean weight ± SD = 20.6 kg ± 2.6 kg), the lambs were euthanised by barbiturate overdose then the cadavers were transported from the farm to the dissecting room (~ 5 min). Oropharyngeal swabs were taken using cotton-tipped swabs (Swab Plain Wood Cotton Tip Sterile (710-0181), Copan, Italy). To prevent oral contamination, the swabs were stored in protective plastic sheaths from which the swab could be advanced and retracted once it was positioned at the sampling site. Swabs were then transferred into a new plastic sheath and stored on ice.

The ventral aspect of the neck was shaved, and a sterile scalpel used to incise through the skin and subcutaneous tissues to expose the ventral surface of the trachea. A sampling site was identified on the exposed ventral surface, and the trachea cranial to this site was completely closed off by both string ligature and clamp placement. The selected sampling site was then heat seared, and 50 ml of sterile phosphate-buffered saline (PBS) was injected through the seared section into the tracheal lumen. The head and neck were oriented such that the PBS would flow caudally down the thorax. A second clamp was immediately placed caudal to the site of injection to prevent backflow, leakage and potential contamination. The lamb cadavers were then tipped so that the PBS would run caudally into their lungs and then tipped back again so that the fluid would collect in the tracheal lumen immediately caudal to the position of the second clamp. A sampling site identified on the ventral surface of the trachea was seared, and a needle and syringe were used to collect the pooled fluid. On average, 4 ± 1.7 ml (mean ± SD) of lung fluid was collected per animal. Lung fluid was stored on ice until further processing. Oropharyngeal swabs were sterilely cut into 500 μl PBS. Lung fluids were centrifuged at 13,000*g* for 5 min. The supernatant was removed, and the pellets were resuspended in 500 μl PBS. The oropharyngeal swabs and lung fluids were stored at −80 °C until DNA extraction.

### DNA extraction, amplification and sequencing

DNA extractions using the PowerSoil DNA isolation Kit (Mo Bio, Carlsbad, USA) and quantitative PCR (qPCR) using the 16S rRNA gene qPCR primers UniF340 (5-ACTCCTACGGGAGGCAGCAGT-3) and UniR514 (5-ATTACCGCGGCTGCTGGC-3) were performed as described previously [15]. Extraction kit reagent controls, consisting of reagent-only extractions, were produced for every day DNA extractions were performed. PBS controls were created by extracting DNA from 500 μl of the PBS which had also been used to wash out the lamb lungs. A mock community control was included which has been described previously [15].

A nested PCR reaction was used to produce amplicons for sequencing; this technique was chosen to reduce PCR bias caused by barcoded primers [18]. The first round of PCR amplified the V1-V4 16S hypervariable regions using the primers 28F (5-GAGTTTGATCNTGGCTCAG-3) and 805R (5-GACTACCAGGGTATCTAATC-3). The conditions were 94 °C for 2 min followed by 20 cycles of 94 °C for 1 min, 55 °C for 45 s and 72 °C for 1.5 min followed by a final extension step of 72 °C for 20 min. Clean-up was performed using the AMPure XP PCR purification system (Beckman Coulter, Brea, USA).

In a previous study, we found that PCR bias in high template samples could be reduced by diluting amplicons from the first round of PCR to a similar concentration to those of lung fluid samples [15]. Therefore, in this study, we used our qPCR values to calculate the dilutions needed to achieve this. The second round of PCR used the barcoded V2-V3 primers 104F (5-GGCGVACGGGTGAGTAA-3) and 519R (5-GTNTTACNGCGGCKGCTG-3). The dilutions and barcoded primers used for each sample can be found in Additional file 1. The PCR conditions were 98 °C for 30 s followed by 20 cycles of 98 °C for 10 s, 67 °C for 30 s and 72 °C for 10 s followed by a final extension step of 72 °C for 2 min. The amplicons were again purified using the AMPure XP PCR purification system.

### Bioinformatic and statistical analysis

Samples were sequenced via either Illumina Miseq or Hiseq runs (Illumina, San Diego, USA) (Additional file 1) producing 250 base pair paired-end reads. Cutadapt [19] was used to remove primers. Quality control and taxonomic assignment of sequences was carried out within mothur [20] following a protocol created by the mothur developers [21], adjusted to suit our dataset [15]. Sequences were subsampled before statistical analysis. The sequencing error rate, principal coordinate analysis (PCOA) graphs, analyses of molecular variance (AMOVAs), Good’s coverage analyses [22], richness (Chao 1 index) and diversity (inverse Simpson index) calculations and indicator analyses [23] were all calculated within mothur. Clustering of microbial communities into metacommunities was also carried out within mothur using a probabilistic modelling technique based upon work by Holmes et al. [24]. The significance of differences between the diversity and richness of groups was calculated using either the two-sample *t* test (normal data) or the Mann-Whitney U test (non-normal data) within Minitab 16 for Windows (Minitab, Coventry, UK). Heatmaps were constructed in R Version 3.2.2 [25] using the Vegan [26], RColorBrewer [27], gplots [28] and heatplus [29] packages. Clustering within heatmaps was performed using the Bray-Curtis dissimilarity [30]. Sequences can be accessed via the Bioproject accession number PRJNA317719.

## Results

### Quality assurance of methodology

In total, 11,878,769 sequence reads were produced with an average of 138,125 ± 29,306 per sample (mean ± SD). The sequencing error rate was calculated as 0.35%. The oropharyngeal swab sample from lamb 12773 was found to have very low read numbers and was therefore discarded from statistical analyses, as was its corresponding lung fluid sample. A total of 1061 OTUs were identified (Additional file 2) which were reduced to 750 after subsampling. All Good’s coverage values were > 0.999 indicating that at least 99.9% of the bacteria present in our samples are likely to have been identified. The most abundant bacterial OTUs from extraction kit reagent-only controls are listed in Table 1. The similarity of the OTUs found on 25 and 26 March 2015 is likely due to the fact that the same lot of extraction kit was used. Upon examining our data, we found that lung fluid samples clustered by when they were processed (Additional file 3). Samples sequenced via Miseq and Hiseq underwent DNA extraction and PCR amplification separately. We identified two OTUs which were significantly indicative (*P* < 0.05) of samples from either the Hiseq or Miseq run which were also present in all lung fluid samples from the run they were indicative of: OTU 4 (*Pseudomonas*) and OTU 112 (Yaniellaceae). These OTUs are likely due to contamination and were therefore removed prior to analysis.

**Table 1:** Bacterial OTUs found to be > 5% abundant in extraction kit reagent controls

Date of DNA extraction	OTUs	Abundance (%)
17 July 2014	*Aerococcus*	14
	Dermabacteraceae	12
	*Micrococcus*	10
	*Enhydrobacter*	9
	*Leuconostoc*	6
	*Kocuria*	6
	*Actinomyces*	6
25 March 2015	*Methylobacterium komagatae*	65
	Ruminococcaceae	11
	*Methylobacterium*	6
26 March 2015	*Methylobacterium komagatae*	67
	*Methylobacterium*	6

### Lamb oropharyngeal swabs cluster into two distinct community types

Oropharyngeal swabs were taken from 40 lambs. Using the Laplace approximation, it was found that the swabs could be partitioned into two separate groups based upon the types of bacteria present. These appeared to correspond to either oropharyngeal-type (partition 1) or rumen-type (partition 2) bacteria (Additional file 4). The oropharyngeal-type communities were dominated by the OTUs Pasteurellaceae (22%), *Mannheimia* (14%), *Fusobacterium* (11%), *Bibersteinia trehalosi* (8%), Neisseriaceae (7%), *Moraxella* (6%) and *Bibersteinia* (5%). The rumen-type communities were dominated by the OTUs *Prevotella* (36%), Clostridiales (11%), Ruminococcaceae (7%), Lachnospiraceae (6%) and *Butyrivibrio* (6%).

The richness (Chao 1: non-normal data) and diversity (inverse Simpsons: normal data) of the partitions were compared. There was no significant difference in richness or diversity between the rumen-type partition and the oropharyngeal-type partition.

### Dichotomous oropharyngeal microbiota are associated with different lung community structures

**Fig. 1:**
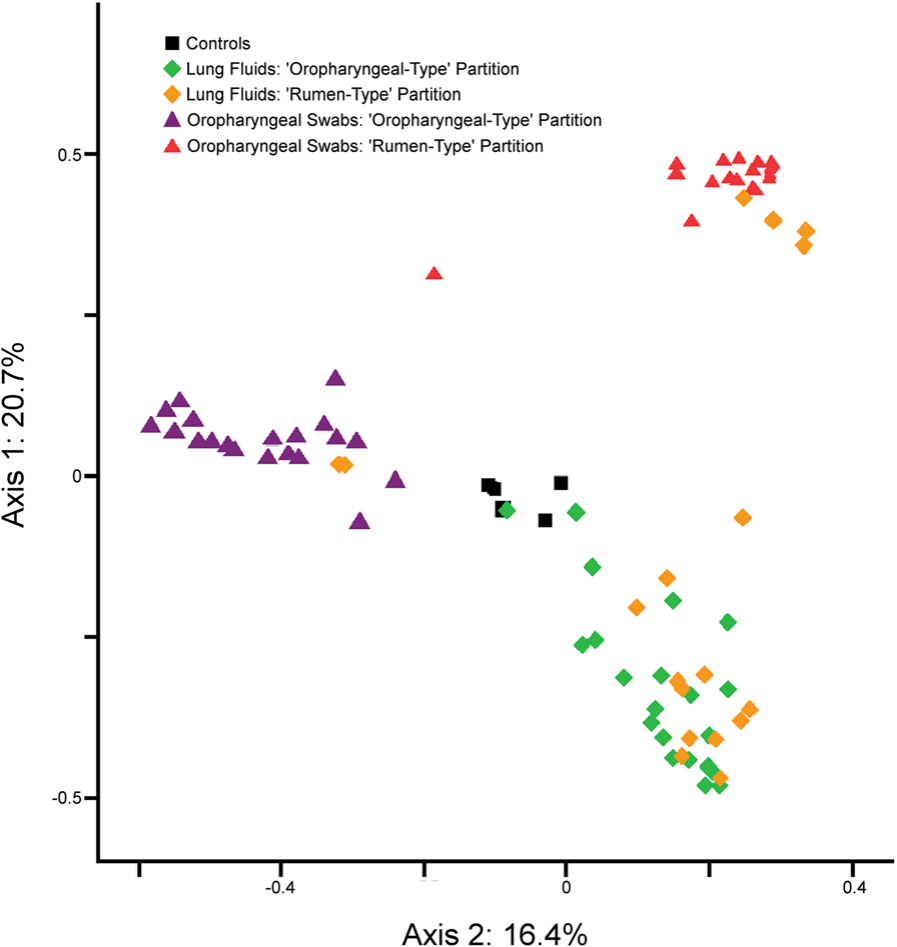
PCOA graph showing the relatedness of upper aerodigestive tract samples from lambs partitioned into two groups using the Laplace approximation. Lung fluids belonging to the same animals were partitioned into the same groups. Lung fluid partitions clustered significantly separately by AMOVA (*P* = 0.016) as did oropharyngeal swabs (*P* < 0.001). Controls are PBS and extraction kit reagent controls

**Fig. 2:**
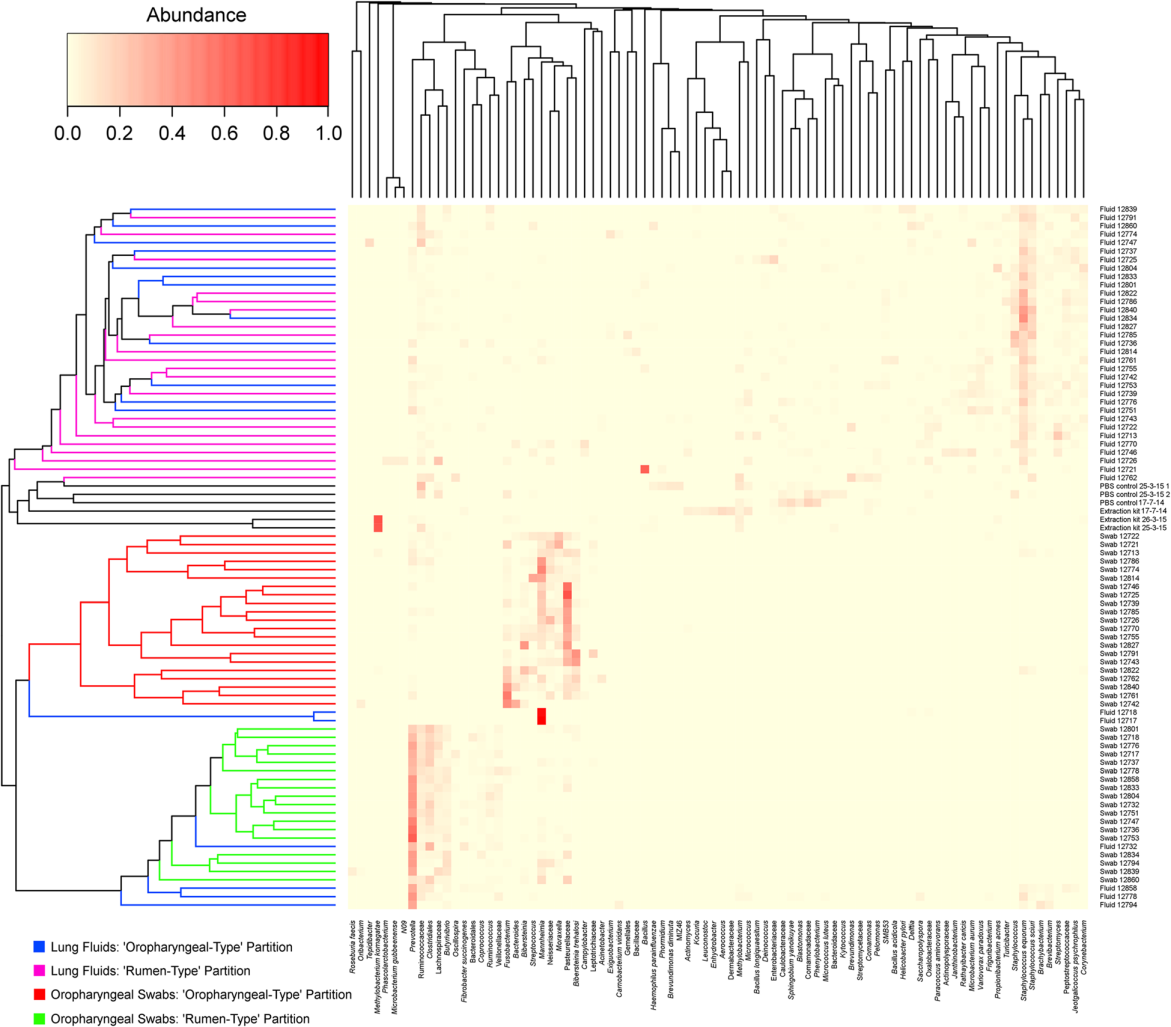
Heatmap of OTUs found in lamb lung fluids, oropharyngeal swabs, extraction kit reagent and PBS controls. OTUs were included when they were > 5% abundant in at least one sample. Oropharyngeal swabs partitioned into rumen-like bacterial communities are indicated by green whereas those which were upper aerodigestive tract-like are indicated by a red line. The lung fluid samples from the oropharyngeal-like animals are indicated by blue whereas those from the rumen-type animals are indicated by pink

The most common OTUs found in the lung fluid samples were *Staphylococcus equorum* (13%), *Staphylococcus sciuri* (6%), *Mannheimia* (5%) and *Prevotella* (5%). Using the Laplace approximation, lung fluids did not cluster into more than one group. Lung fluids were then manually partitioned into the same groups as swabs. A significant difference in bacterial community structure was found between these groups (AMOVA: *P* = 0.016), and a small number of OTUs were found to be significantly different between the two groups. *Prevotella* (*P* = 0.03) and *Sphingobium* (*P* = 0.039) were significantly indicative of lambs from which rumen-type swabs were derived whereas *Paracoccus aminovorans* (*P* = 0.036) was indicative of lambs from which oropharyngeal-type swabs were derived. Figures 1 and 2 contain visual representations of sample clustering.

We compared the proportions of the dominant OTUs in rumen-type swabs with their corresponding proportions in lung samples. On average, these OTUs were found in the following proportions in lung samples: *Prevotella* (5%), Clostridiales (2%), Ruminococcaceae (3%), Lachnospiraceae (1%) and *Butyrivibrio* (1%).

### The presence of a lung-specific microbiota

**Fig. 3:**
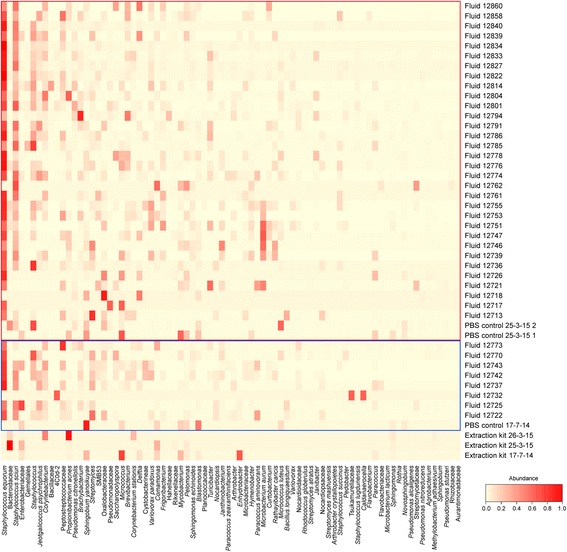
Heatmap of bacterial OTUs found to be more indicative of lamb lung fluids than oropharyngeal swabs (*P* < 0.05). Fluid and PBS samples from which DNA was extracted on specific dates are surrounded by coloured lines: 17 July 2014 (blue) and 25 March 2015 (red). DNA extractions carried out on 26 March 2015 only comprised oropharyngeal swabs which are not included in this figure

Indicator species analysis determined that several OTUs were significantly more indicative of the lungs than of oropharyngeal swabs (Table 2). It is likely that reagent contamination will have had more of an impact on the lung fluid samples than on the oropharyngeal swabs, due to their lower biomass. However, when examining the indicative OTUs, the majority of samples were not found to contain the same proportions of these OTUs as the PBS controls processed alongside them (Fig. 3). Of the indicative OTUs, by far the most common was *Staphylococcus equorum* which constituted, on average, 13.3% of the total bacteria present in lung fluids and which was only present in low numbers in controls and oropharyngeal swabs.

**Table 2:** OTUs significantly more indicative of lung fluids than oropharyngeal swabs

Taxonomy	*P* value	Average proportion in lung fluids (mean ± SD) (%)	Average proportion in oropharyngeal swabs (mean ± SD) (%)	Highest proportion in PBS controls (%)
*Brachybacterium*	0.006	1.0 ± 1.7	0.035 ± 0.10	0.022
*Brevibacterium*	0.002	1.2 ± 1.4	0.064 ± 0.24	0
*Corynebacterium*	< 0.001	1.9 ± 2.3	0.065 ± 0.19	0.044
*Delftia*	< 0.001	0.80 ± 1.7	0 ± 0	0
Enterobacteriaceae	0.023	0.65 ± 2.6	0.0063 ± 0.029	2.2
*Frigoribacterium*	0.021	0.79 ± 1.4	0.077 ± 0.31	0
*Janthinobacterium*	0.01	0.57 ± 1.4	0.0023 ± 0.0068	0
*Jeotgalicoccus psychrophilus*	0.008	1.6 ± 2.1	0.040 ± 0.10	0
*Microbacterium aurum*	0.047	1.2 ± 2.8	0.0045 ± 0.013	0
*Micrococcus*	0.017	0.77 ± 1.6	0.0080 ± 0.029	4.4
Oxalobacteraceae	< 0.001	0.96 ± 1.5	0.012 ± 0.043	3.0
*Pelomonas*	< 0.001	0.65 ± 1.1	0.00057 ± 0.0036	2.0
Peptostreptococcaceae	0.006	1.8 ± 2.2	0.050 ± 0.11	0.044
*Propionibacterium acnes*	< 0.001	0.84 ± 2.3	0.0040 ± 0.020	1.6
*Pseudomonas citronellolis*	< 0.001	0.51 ± 1.1	0 ± 0	1.0
*Rathayibacter caricis*	0.016	0.58 ± 1.2	0.0057 ± 0.021	0
*Saccharopolyspora*	0.009	0.52 ± 1.2	0.0011 ± 0.0071	0
*SMB53*	< 0.001	0.71 ± 1.3	0.0045 ± 0.018	0
*Sphingobium yanoikuyae*	< 0.001	0.53 ± 0.56	0 ± 0	13
*Staphylococcus*	< 0.001	3.9 ± 5.4	0.060 ± 0.18	8.6
*Staphylococcus equorum*	< 0.001	13.3 ± 9.6	0.32 ± 0.97	0.044
*Staphylococcus sciuri*	< 0.001	6.4 ± 5.3	0.18 ± 0.59	2.0
*Streptomyces*	< 0.001	2.0 ± 3.8	0.025 ± 0.096	0
*Turicibacter*	0.016	1.0 ± 1.8	0.0074 ± 0.021	0
*Variovorax paradoxus*	0.011	1.2 ± 1.2	0.024 ± 0.060	0.022

OTUs were significantly more (*P* < 0.05) indicative of lamb lung fluids than oropharyngeal swabs and were on average > 0.5% abundant in lung fluids

## Discussion

Sheep are commonly used as large animal models of the respiratory system due to the physiological and immunological similarities of their lungs to those of humans [10, 11, 31, 32]. We have previously used sheep to study both the extent of variation in the lung microbiota [15] and the direct and remote changes in the lung microbiota caused by localised *P*. *aeruginosa* infection and antibiotic treatment [14]. As the sheep is an important agricultural animal, studies of its respiratory microbial communities may also be of interest from an animal health perspective.

It has previously been demonstrated that microaspiration of microbes from the upper aerodigestive tract is common in humans and can lead to an inflammatory phenotype [33]. When microbial communities from healthy human lungs are characterised, they are often found to contain microbes associated with the upper aerodigestive tract [34]. The healthy human lung microbiota is thought to be formed predominantly from the neutral dispersal of these upper aerodigestive tract microbes into the lungs rather than by the differential growth of lung-adapted microbial communities [35]. We sought to identify whether this was also the case in sheep.

Sheep oropharyngeal swabs could be partitioned into two separate groups which were predominantly composed of OTUs identified as bacteria which are well known members of either the rumen (*Prevotella*, Clostridiales, Ruminococcaceae, Lachnospiraceae and *Butyrivibrio* [36–38]) or respiratory tract microbiotas (Pasteurellaceae, *Mannheimia*, *Fusobacterium*, *Bibersteinia trehalosi*, Neisseriaceae, *Moraxella* and *Bibersteinia* [39–41]). These bacteria were also detected in a previous study examining sheep buccal swabs [42].

It is not possible to identify whether this dichotomy reflected recent rumination or some stochastic post-mortem leakage of rumen fluid into the oropharynx in some individuals. The lambs during this study were not weaned but were at an age when it is expected that they all would be regularly supplementing their diet with grass and would be ruminating.

Regardless of the drivers of this oropharyngeal dichotomy, the microbial communities found in the lungs were very different to those found in both the rumen- and oropharyngeal-type swabs. A large number of bacterial OTUs were found to be significantly more abundant in lung fluids in comparison to oropharyngeal swabs, including *Staphylococcus equorum* which was by far the most common bacterial OTU found. Several OTUs which are commonly associated with the rumen were also identified in lung fluids. Our lung fluid samples will have been more affected by reagent contamination than the oropharyngeal swabs due to the lower quantity of bacterial DNA present [43]. However, the microbial communities found in lung fluids did not reflect the bacteria found in reagent-only controls processed on the same day, so the presence of bacteria in the lamb lung cannot be attributed purely to sample contamination nor can it be attributed to disease as no lambs showed clinically overt signs of respiratory illness during the study.

There are several reasons why the microbes found in lamb lungs might not reflect those found in the upper aerodigestive tract to the same extent as is found in humans. Sheep have evolved to cope with rumination and thereby may have more efficient anatomical barriers to microaspiration [44]. Physiological and anatomical differences such as the horizontal disposition of the lungs, increased nasal breathing and increased saliva production [45, 46] may also contribute.

## Conclusions

In this study, we examined oropharyngeal swab and lung fluid samples taken from healthy lambs to characterise the bacterial communities present and to assess the impact of rumination on these communities. We found that the oropharyngeal swabs were dominated by either rumen-type or oropharyngeal-type microbial communities. We also found that lung bacteria did not greatly resemble either rumen- or oropharyngeal-type swabs and identified several bacterial OTUs which were more indicative of lung fluids. The lungs did contain several rumen-associated bacteria which may indicate that there is a certain degree of microaspiration of ruminal contents in lambs.

Sheep are not human, but the opportunities that they, and other large animals, present offer valuable insights into the dynamic relationship of the upper aerodigestive and lower respiratory tract microbiota in health. In the future, their value may extend to developing an understanding of the factors that predispose the upper aerodigestive tract microbiota towards disease in the lower respiratory tract.

## Additional files

Additional file 1: Dataset S1. Sample processing data for all samples. (XLSX 18 kb)

Additional file 2: Dataset S2. Full list of bacterial OTUs and taxonomies. (XLSX 370 kb)

Additional file 3: Figure S1. Heatmap of OTUs found in lamb lung fluids, oropharyngeal swabs, PBS and extraction kit reagent-only controls. (DOCX 716 kb)

Additional file 4: Table S1. OTUs responsible for partitioning of lamb oropharyngeal swabs into two groups (using Laplace value). (DOCX 16 kb)
