# Supplementary material for: Comparing microbiotas in the upper aerodigestive and lower respiratory tracts of lambs
Source: Microbiome. 2017 Oct 27;5:145. doi: 10.1186/s40168-017-0364-5 (PMC5658956; doi:10.1186/s40168-017-0364-5)
Supplement: Supplementary file 3 — Figure S1. Heatmap of OTUs found in lamb lung fluids, oropharyngeal swabs, PBS and extraction kit reagent-only controls. (DOCX 716 kb) [file 40168_2017_364_MOESM3_ESM.docx]

**Figure S1: Heatmap of OTUs found in lamb lung fluids, oropharyngeal swabs, PBS and extraction kit reagent only controls. OTUs were included when they were > 5% abundant in at least one sample. Samples whose DNA was extracted on separate days are indicated by the following colours on the hierarchical clustering dendrogram: 17^th^ July 2014 = blue; 25^th^ March 2015 = red; 26^th^ March 2015 = green.**

**
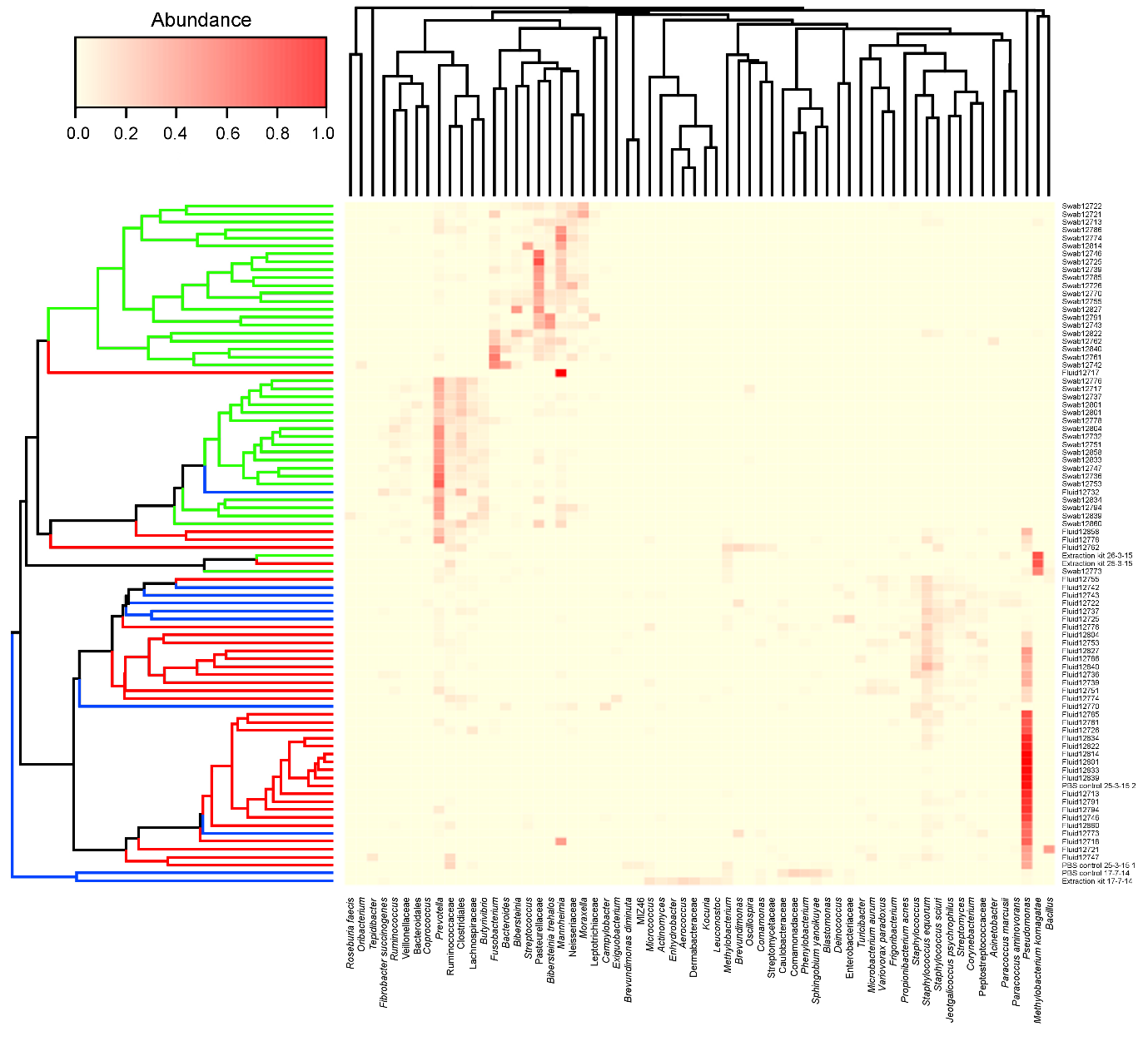
**
