# Supplementary material for: Comparing microbiotas in the upper aerodigestive and lower respiratory tracts of lambs
Source: Microbiome. 2017 Oct 27;5:145. doi: 10.1186/s40168-017-0364-5 (PMC5658956; doi:10.1186/s40168-017-0364-5)
Supplement: Supplementary file 4 — OTUs responsible for partitioning of lamb oropharyngeal swabs into two groups (using Laplace value). (DOCX 16 kb) [file 40168_2017_364_MOESM4_ESM.docx]

**Table S1: OTUs responsible for partitioning of lamb oropharyngeal swabs into two groups (using Laplace value).**

| Taxonomy | Indicator groups | P value | |
| --- | --- | --- | --- |
| *Bacteroides* | Partition 1 | <0.001 |  |
| *Bibersteinia trehalosi* | Partition 1 | <0.001 |  |
| *Campylobacter rectus* | Partition 1 | <0.001 |  |
| *Leptotrichia* | Partition 1 | <0.001 |  |
| *Moraxella* | Partition 1 | <0.001 |  |
| *Porphyromonas* | Partition 1 | <0.001 |  |
| *Streptococcus* | Partition 1 | <0.001 |  |
| *Streptococcus minor* | Partition 1 | <0.001 |  |
| *Kingella* | Partition 1 | 0.003 |  |
| *Moraxella ovis* | Partition 1 | 0.003 |  |
| Pasteurellaceae | Partition 1 | 0.003 |  |
| Comamonadaceae | Partition 1 | 0.004 |  |
| Cardiobacteriaceae | Partition 1 | 0.005 |  |
| *Corynebacterium* | Partition 1 | 0.005 |  |
| Lactobacillales | Partition 1 | 0.005 |  |
| *Tannerella* | Partition 1 | 0.005 |  |
| BD1-5 | Partition 1 | 0.006 |  |
| *Bibersteinia* | Partition 1 | 0.006 |  |
| *Fusobacterium* | Partition 1 | 0.007 |  |
| *Mannheimia* | Partition 1 | 0.007 |  |
| *Streptococcus agalactiae* | Partition 1 | 0.007 |  |
| *Actinomyces hyovaginalis* | Partition 1 | 0.008 |  |
| *Aggregatibacter* | Partition 1 | 0.008 |  |
| Caulobacteraceae | Partition 1 | 0.008 |  |
| *Methylobacterium komagatae* | Partition 1 | 0.008 |  |
| Neisseriaceae | Partition 1 | 0.008 |  |
| *Conchiformibius kuhniae* | Partition 1 | 0.009 |  |
| *Variovorax paradoxus* | Partition 1 | 0.009 |  |
| *Dietzia* | Partition 1 | 0.01 |  |
| *Hylemonella* | Partition 1 | 0.01 |  |
| Streptococcaceae | Partition 1 | 0.01 |  |
| *Lautropia* | Partition 1 | 0.011 |  |
| *Frigoribacterium* | Partition 1 | 0.014 |  |
| Micrococcaceae | Partition 1 | 0.014 |  |
| Peptostreptococcaceae | Partition 1 | 0.014 |  |
| *Actinomyces* | Partition 1 | 0.016 |  |
| *Streptobacillus* | Partition 1 | 0.016 |  |
| *Staphylococcus* | Partition 1 | 0.019 |  |
| *Staphylococcus sciuri* | Partition 1 | 0.024 |  |
| *Campylobacter* | Partition 1 | 0.025 |  |
| *Peptostreptococcus anaerobius* | Partition 1 | 0.026 |  |
| Propionibacteriaceae | Partition 1 | 0.026 |  |
| *Brachybacterium* | Partition 1 | 0.027 |  |
| Erysipelotrichaceae | Partition 1 | 0.029 |  |
| Fusobacteriaceae | Partition 1 | 0.029 |  |
| *Parvimonas* | Partition 1 | 0.029 |  |
| *Streptococcus equi* | Partition 1 | 0.032 |  |
| *Facklamia* | Partition 1 | 0.037 |  |
| *Microvirgula* | Partition 1 | 0.04 |  |
| *Clavibacter michiganensis* | Partition 1 | 0.043 |  |
| *Sphingomonas echinoides* | Partition 1 | 0.043 |  |
| *Staphylococcus equorum* | Partition 1 | 0.045 |  |
| *Acinetobacter venetianus* | Partition 1 | 0.046 |  |
| *Acinetobacter lwoffii* | Partition 1 | 0.047 |  |
| *Arthrobacter* | Partition 1 | 0.047 |  |
| Bacteroidales | Partition 2 | <0.001 |  |
| *Blautia* | Partition 2 | <0.001 |  |
| *Butyrivibrio* | Partition 2 | <0.001 |  |
| Carboxydocellaceae | Partition 2 | <0.001 |  |
| *CF231* | Partition 2 | <0.001 |  |
| Clostridiales | Partition 2 | <0.001 |  |
| *Clostridium* | Partition 2 | <0.001 |  |
| *Coprococcus* | Partition 2 | <0.001 |  |
| Coriobacteriaceae | Partition 2 | <0.001 |  |
| *Desulfovibrio D168* | Partition 2 | <0.001 |  |
| *Eggerthella* | Partition 2 | <0.001 |  |
| *Faecalibacterium* | Partition 2 | <0.001 |  |
| *Fibrobacter succinogenes* | Partition 2 | <0.001 |  |
| Lachnospiraceae | Partition 2 | <0.001 |  |
| Mogibacteriaceae | Partition 2 | <0.001 |  |
| *Mogibacterium* | Partition 2 | <0.001 |  |
| *Moryella* | Partition 2 | <0.001 |  |
| *Moryella indoligenes* | Partition 2 | <0.001 |  |
| *Oscillospira* | Partition 2 | <0.001 |  |
| *Oscillospira guilliermondii* | Partition 2 | <0.001 |  |
| *p-75-a5* | Partition 2 | <0.001 |  |
| Paraprevotellaceae | Partition 2 | <0.001 |  |
| *Persicobacter* | Partition 2 | <0.001 |  |
| *Prevotella* | Partition 2 | <0.001 |  |
| Prevotellaceae | Partition 2 | <0.001 |  |
| *Prevotella ruminicola* | Partition 2 | <0.001 |  |
| *Pseudobutyrivibrio* | Partition 2 | <0.001 |  |
| *Pyramidobacter* | Partition 2 | <0.001 |  |
| *RFN20* | Partition 2 | <0.001 |  |
| Ruminococcaceae | Partition 2 | <0.001 |  |
| *Ruminococcus flavefaciens* | Partition 2 | <0.001 |  |
| S24-7 | Partition 2 | <0.001 |  |
| *SHD-231* | Partition 2 | <0.001 |  |
| *Shuttleworthia* | Partition 2 | <0.001 |  |
| *Succiniclasticum* | Partition 2 | <0.001 |  |
| TTA_B6 | Partition 2 | <0.001 |  |
| Veillonellaceae | Partition 2 | <0.001 |  |
| *YRC22* | Partition 2 | <0.001 |  |
| YS2 | Partition 2 | <0.001 |  |
| *Desulfovibrio* | Partition 2 | 0.002 |  |
| *Ruminococcus* | Partition 2 | 0.004 |  |
| Fibrobacteraceae | Partition 2 | 0.006 |  |
| *Treponema* | Partition 2 | 0.006 |  |
| RF39 | Partition 2 | 0.007 |  |
| Chloroherpetales | Partition 2 | 0.009 |  |
| Thermogemmatisporales | Partition 2 | 0.012 |  |
| Saprospirae | Partition 2 | 0.013 |  |
| *L7A_E11* | Partition 2 | 0.014 |  |
| SSW63Au | Partition 2 | 0.015 |  |
| *Anaerofustis* | Partition 2 | 0.017 |  |
| Christensenellaceae | Partition 2 | 0.018 |  |
| *Elizabethkingia* | Partition 2 | 0.022 |  |
| *Anaerostipes* | Partition 2 | 0.023 |  |
| *Bosea* | Partition 2 | 0.024 |  |
| Spirochaetaceae | Partition 2 | 0.024 |  |
| *Kurthia* | Partition 2 | 0.029 |  |
| BS11 | Partition 2 | 0.031 |  |
| Methylacidiphilae | Partition 2 | 0.033 |  |
| *Anaeroplasma* | Partition 2 | 0.034 |  |
| Rhodothermales | Partition 2 | 0.036 |  |
| *BF311* | Partition 2 | 0.044 |  |
| Deferribacterales | Partition 2 | 0.045 |  |
| Desulfovibrionaceae | Partition 2 | 0.048 |  |
| *Selenomonas ruminantium* | Partition 2 | 0.048 |  |

**The OTUs sorted into partition 1 are more oropharyngeal-like whereas those sorted into partition 2 are more rumen-like. The OTUs which were significantly indicative of either partition 1 or 2 were calculated using the indicator command within mothur.**
